# Geographical information dataset “geosynthetics in coastal protection of the South-East Baltic”

**DOI:** 10.1016/j.dib.2021.107693

**Published:** 2021-12-08

**Authors:** Dmitry Domnin, Eugeny Burnashov

**Affiliations:** a Shirshov Institute of Oceanology, Russian Academy of Sciences, Moscow 117997, Russia; b SBI KO “Baltberegozashchita”, Svetlogorsk 238560, Russia

**Keywords:** Geosynthetics, Geotextiles, Coastal protection, GIS, Remote data, Baltic Sea

## Abstract

The database provides information on coastal protection structures (containing geosynthetic materials) located on the coast of the South-East Baltic, in the Kaliningrad Oblast (Russia) and the Pomeranian Voivodeship (Poland). The fragments of geosynthetics may enter the environment due to partial destruction and operational losses and become a new type of coastal pollution. The database contains the following sections: the tabular data about coastal protecting structures [ProtectingStructures_tab.xlsx]; the point vector geodata about these structures [ProtectingStructures_pnt.kmz] and used geosynthetic materials; the satellite images and photos [ProtectingStructures_images.pdf] demonstrated the general and close-up location of the coastal protecting structures in the satellite image, as well as their seeming. Information was collected during the ERANET-RUS_Plus joint project EI-GEO, ID 212 (RFBR 18-55-76002 ERA_a, BMBF 01DJ18005).

## Specifications Table

**Table:** 

Subject	Environmental Science, Ecology, Earth Science
Specific subject area	Potential contamination of the marine environment by geosynthetic material debris
Type of data	Tabular data, vector geodata, images
How data were acquired	The location of the objects and their metric characteristics were obtained using satellite images. Information on the use of geosynthetics was taken from archival and published sources. The authors took the actual images of the structures and used the archival and open published sources. Post-processing: MS Excel, QuantumGIS
Data format	raw
Parameters for data collection	Spatial information on location and metric characteristics was obtained from satellite images (SAS.Planet service (http://www.sasgis.org/category/sasgis/) with a maximum resolution of 2.7 m/pixel.
Description of data collection	In total, 16 coastal protection structures in the Kaliningrad Oblast (Russia) and 17 ones in Pomeranian Voivodeship (Poland) were identified as potential sources of geosynthetic pollution at the open coast of the South-East Baltic. For each structure, their type, location (including settlement and coordinates), year of construction (or last reconstruction), type of geosynthetics used in it, its length, the width of the beach in front of the structure are determined.
Data source location	Institution: Shirshov Institute of Oceanology, Russian Academy of Sciences Country (region): Russian Federation (Kaliningrad Oblast) Latitude and longitude of central points for coastal protection structures: The study area rectangle (sandy beaches at the non-tidal shore of the Kaliningrad Oblast of Russia and Pomeranian Voivodeship of Poland, in the South-Eastern Baltic) is described by coordinates of the left down corner (N 54.367006, E 16.870667) and the right top corner (N 54.972483, E 20.509458).
Data accessibility	To the Repository. Repository name: Mendeley Data Data identification number: DOI:10.17632/jbd4r9vwpb.2 Direct URL to data: https://data.mendeley.com/datasets/jbd4r9vwpb/2

## Value of the Data

• Geosynthetic materials are widely used in various structures, including coastal protection, anti-landslide and anti-erosion structures. In case of damage or prolonged operation, geosynthetics debris can pollute the marine environment.
• A dataset presents the information on coastal protection structures containing geosynthetic materials. These structures could be the potential sources for geosynthetic debris emission into the marine environment of the South-East Baltic.
• Researchers, beach managers and practitioners may use these data.
• The data can be used further as reference data to estimate the progress in beach cleaning for this part of the Baltic shores or comparison with other shores. The data should be used to identify the sources of geosynthetic contamination related to the destruction of coastal protection structures and construction activities at the shore, which helps engineers avoid it in the future.

## 1 Data Description

The data is collected in some separate data files, which have open access with the article and to repository:

- tabular data: spreadsheets (∗.xlsx) (to repository [1]);
- vector geodata: imtaractive map data (∗.kmz) (to repository [1]),
- images: satellite images and photos embedded in the PDF document (∗.pdf) (to repository [1]).

Spreadsheets (named “ProtectingStructures_tab.xlsx”) contain the coastal protection structures located on the sea coast of the South-East Baltic. Sheet 1 (named “ProtectingStructuresSEB”) shows a list of coastal protection structures in the South-Eastern Baltic that contain geosynthetics and their characteristics. It has 10 columns (Table 1): the number (the conventional two-level number assigned to the structure); the type (type of structure); the location (closest settlement to which the structure is located); the country (country where the structure is located); Building_Reconstruction_year (the year of building or last reconstruction of the structure); Geosyntetic_type (type of geosynthetic material used in the structure); Length_m (length of the structure in m); Width_beach_m (width of the beach in front of the structure, range, m); Lat (latitude, °); Lon (longitude, °). Sheet 2 (named “Legend”) shows the legend described above.

**Table 1:** Fragment of the table “ProtectingStructures_tab.xlsx” shows a list of coastal protection structures in the South-Eastern Baltic that contain geosynthetics and their characteristics.

Number	Type	Location	Country	Building_ Reconstruction_ year	Geosyntetic_ type	Length, m	Width_ beach, m	Lat°	Lon°
1.01	Chain-like concrete cover of foredune	Sobieszewo	PL	2006	geotextile (inside the construction)	220	35	54.3670	18.7887
2.04	Gabions	Svetlogorsk	RU	2008	gabion coating (outside the construction)	1400	10	54.9453	20.1413

Intaractive map data [ProtectingStructures_pnt.kmz] is the point vector layer that contains the information about coastal protection structures located on the sea coast of the South-East Baltic. Projected Coordinate System is WGS 1984, UTM Zone 34N, Projection is Transverse Mercator. The attribute table has the columns (Table 2): the number (the conventional two-level number assigned to the structure); the type (type of structure); the location (closest settlement to which the structure is located); the country (country where the structure is located); Building_Reconstruction_year (year of building or last reconstruction of the structure); each type of geosynthetics has a separate column (Geotextile, Gabion_coating, Geocontainers, Geocells, Geomat, PVC_sheet_pile), the absence of geosynthetics is designated as “0”, the presence of the geosynthetics is designated as “1”; Length_m (length of the structure in m); Width_beach_m (width of the beach in front of the structure, range, m); Lat (latitude, °); Lon (longitude, °).

**Table 2:** Fragment of the attribute table of point intaractive map data “ProtectingStructures_pnt.kmz” shows a list of coastal protecting structures that contain geosynthetics and their characteristics.

Number	Type	Location	Country	Building_ Recon- struction_ year	Geotextile	Gabion_ coating	Geocontainers	Geocells	Geomat	Length_ m	Width_ beach_m	Lat°	Lon°
1.02	Gabions	Orłowo	PL	2008	1	1	0	0	1	240	15	54.4791	18.5636
2.11	Complex coastal protecting structure	Kulikovo	RU	2020	1	0	0	0	0	410	35	54.9369	20.3518

**Fig. 1:**
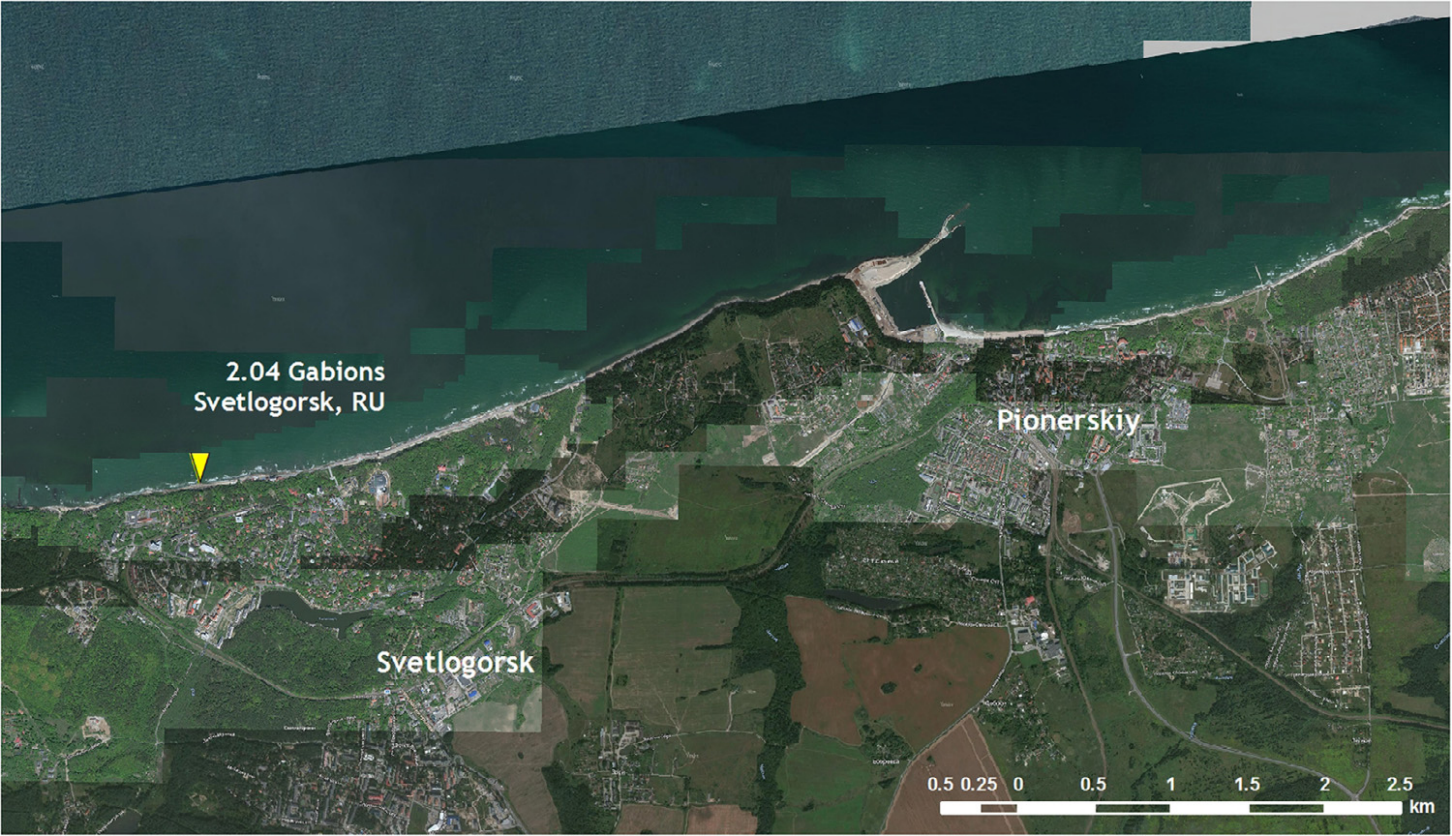
General location of coastal protecting structures (gabions in Svetlogorsk) on the satellite image (Maps Data: Scanex Ltd., Image 2019 DigitalGlobe, Inc. CNES 2013, shooting year: 2019).

**Fig. 2:**
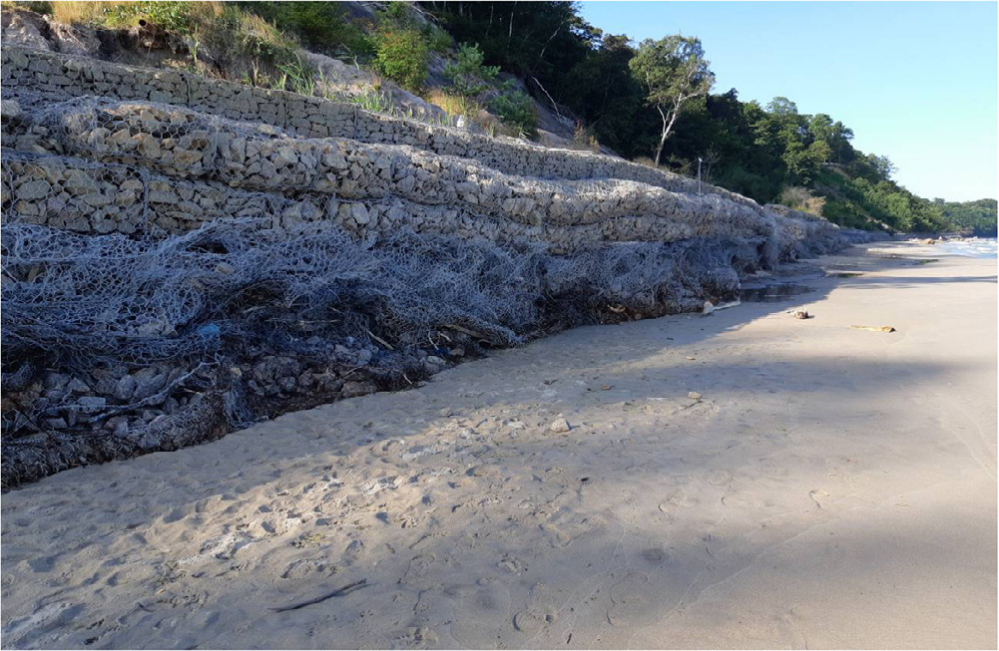
Photo of coastal protecting structure (gabions in Svetlogorsk).

The satellite images and photos are embedded in the PDF document [ProtectingStructures_images.pdf]. They demonstrate the general location (scale 1 : 50 000) of the coastal protection structures in the satellite image (Fig. 1) and their appearance in the photos (Fig. 2).

## 2 Experimental Design, Materials and Methods

Information-Prediction Automatic System (IPAS) was developed for the Baltic Sea shore within the Kaliningrad Oblast in 2005-2006 years and implemented in the State Organization of the Kaliningrad Oblast ``Baltberegozaschita'' (coastal management authority) in 2007. It is used as database for shore protection engineering and a tool for analyses and forecasting of coastal processes. IPAS is regularly used for processing and analysis of data, preparation of analytical notes for the Government of Kaliningrad Oblast and for municipal authorities [2,3].

Modern geosynthetic materials are widely used in various structures, including coastal protection, anti-landslide and anti-erosion structures. In damage or prolonged use, geosynthetics during contact with seawater can break off and enter the Baltic Sea, polluting it [4,5]. The fragments of geosynthetics are transported along the shore and episodically washed out onshore by the same physical mechanisms which form the bach casts [6].

All new structures, including those with geosynthetic material, have been added to the IPAS. According to archival data, it was found that 16 coastal and anti-landslide structures, in which geosynthetic materials were used, are located on the sea coast of the Kaliningrad Oblast. Similar objects (17 ones) were identified on the Polish sea coasts.

Tabular data and vector geodata contained the main characteristics of coastal protection structures (type of structure, year of construction or reconstruction, length of the structure, type of geosynthetics) were obtained from the data of archival materials “Baltbergozaschita” including IPAS's data [3], as well as from open published sources [7], [8], [9], [10], [11], [12].

Spatial data (geographic coordinates, location, width of the beach) were obtained from satellite images fron the GoogleEarth and the YandexMaps via the service SAS.Planet [13].

All indicated structures were built or reconstructed in the period 2004-2020. They belong to the following types (the number of structures is given in brackets): Foredune (foredune wall) (2), Chain-like concrete cover of foredune (1), Gabions (4), Promenade (2), Retaining wall (1), Stair descent (1), Rock armour (1), Stone groin (1), Submerged breakwater (3), Cliff slope strengthening and covering (1), Complex coastal protecting structure (15).

According to Esiukova et al. [4] geosynthetic materials are made from polypropylene (PP), polyester (PET), polyethylene (PE), high-density polyethylene (HDPE), polyamide (nylon), polyvinyl chloride (PVC), and fibreglass. PP and PET are the most widely used materials. The most frequent found debris of geosynthetic materials are related to five types: geotextile, geomat, degraded gabion coating, geocontainers and geocells. Geotextile, geomat and geocells are inside protecting structures, i.e. its serve as an underlying or reinforcing material. Geocontainers and gabion mesh are outside the structure and perform the function of a shell.

The width of the beach in front of the coastal protection structure is an important parameter shows how far the structure is, on average, from the shoreline. Depending on the season, it may vary from complete absence to several tens of meters [14]. In the dataset, the values of beach width were rounded to the nearest 5 m. A value of “0” characterises structures that are entirely submerged or are at the shoreline.

Satellite images demonstrate the general location and close-up location of the coastal protection structures in the satellite image (Fig. 1). Figures were obtained from GoogleEarth and YandexMaps via the SAS.Planet service [13]. Photos of structures were made by the authors or obtained from stock materials (Fig. 2).

**Fig. 3:**
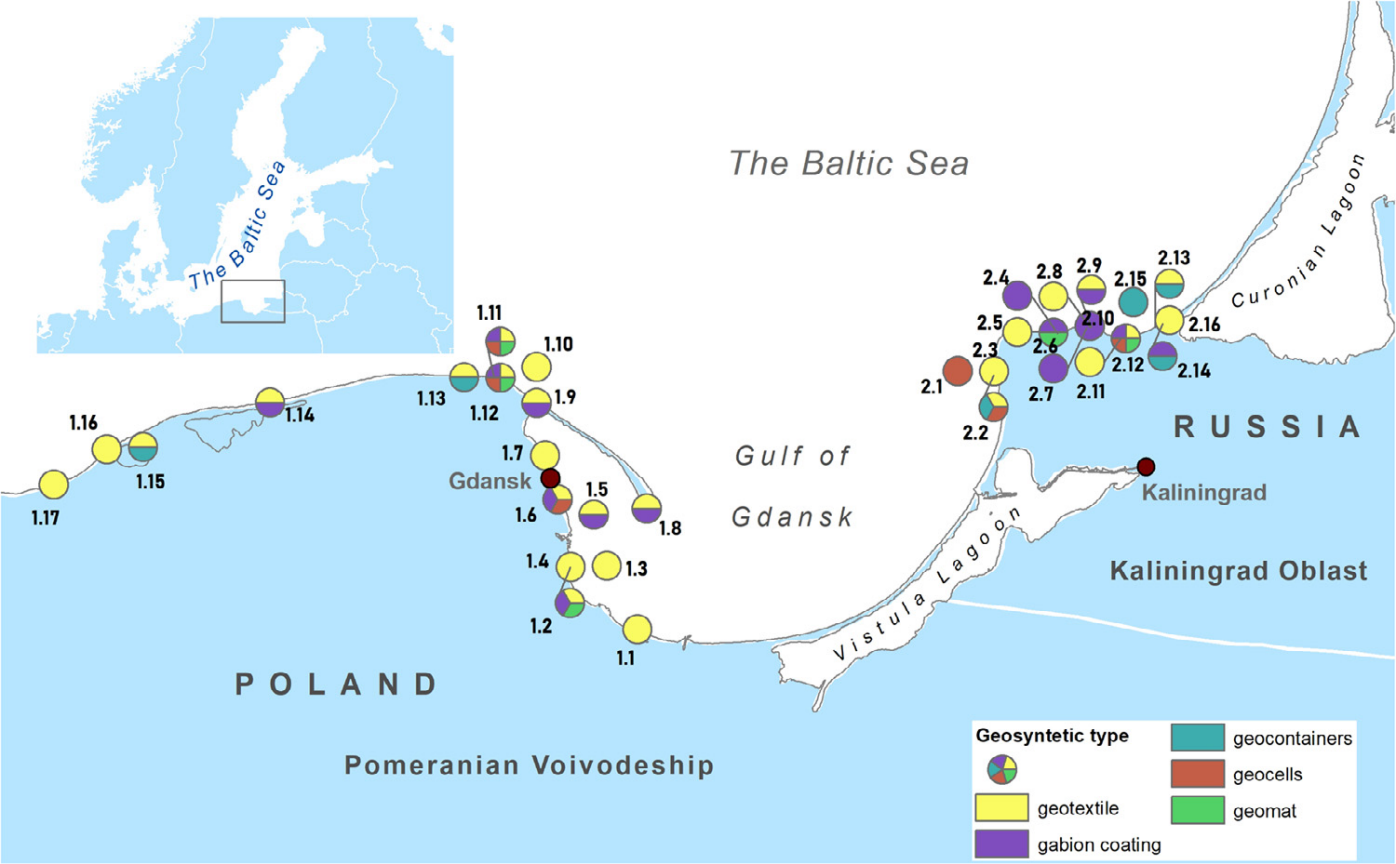
Location of coastal protecting structures on the coast of the South-East Baltic with the indication of types of geosynthetic materials used in their construction.

On the sea coast of the Kaliningrad Oblast (Russia), 16 coastal protection structures contain geosynthetic materials. The total length is about 8 km (Fig. 3). On the eastern coast of Poland, Pomeranian Voivodeship, from the port of Ustka to the Polish-Russian border on the Vistula Spit, there are 17 coastal protection structures with a total length of about 12 km. Almost all identified coastal protection structures were built or reconstructed in the last 20 years.

All these constructions could be a potential source of geosynthetic materials entering the Baltic Sea. The potential primary contaminants can be the remains of gabion braids and scraps of geotextiles used as cushioning material.

## Ethics Statement

It is not relevant to this study.

## CRediT authorship contribution statement

**Dmitry Domnin:** Conceptualization, Methodology, Data curation, Visualization, Writing – original draft. **Eugeny Burnashov:** Resources, Data curation, Writing – original draft.

## Declaration of Competing Interest

The authors declare that they have no known competing financial interests or personal relationships which have or could be perceived to have influenced the work reported in this article.
